# The relative potential contribution of volume load and vascular mechanisms to hypertension in non-dialysis and dialysis chronic kidney disease patients

**DOI:** 10.3389/fcvm.2024.1377887

**Published:** 2024-04-15

**Authors:** Grace Tade, Hon-Chun Hsu, Chanel Robinson, Noluntu Dlongolo, Gloria Teckie, Ahmed Solomon, Patrick Hector Dessein

**Affiliations:** ^1^ Cardiovascular Pathophysiology and Genomics Research Unit, Faculty of Health Sciences, School of Physiology, University of the Witwatersrand, Johannesburg, South Africa; ^2^ Nephrology Unit, Milpark Hospital, Johannesburg, South Africa; ^3^ Rheumatology Unit, Rosebank Hospital, Johannesburg, South Africa; ^4^ Division of Nephrology, Department of Medicine, Chris Hani Baragwanath Hospital and Faculty of Health Sciences, University of Witwatersrand, Johannesburg, South Africa; ^5^ Internal Medicine Department, University of the Witwatersrand, Johannesburg, South Africa

**Keywords:** chronic kidney disease, volume load, stroke volume, total arterial compliance, systemic vascular resistance, blood pressure determinants

## Abstract

**Background:**

Hypertension is highly prevalent and particularly difficult to treat adequately in patients with chronic kidney disease (CKD). The relative contribution of volume overload and vascular mechanisms to blood pressure measures in CKD and whether these effects differ in non-dialysis compared to dialysis patients is unknown.

**Methods:**

We determined the potential impact of volume load (stroke volume) and vascular mechanisms (inverse of total arterial compliance (inv TAC) and systemic vascular resistance (SVR)) on mean and brachial and aortic systolic blood pressures in 67 non-dialysis and 48 dialysis chronic kidney disease (CKD) patients. Relationships were determined in confounder adjusted regression models.

**Results:**

Stroke volume (*p* value = 0.003) was more strongly associated with mean arterial pressure than SVR (*p* value = 0.9) (*p* value for difference = 0.03). When stroke volume and SVR were entered in the same regression model (model *R*^2^^ ^= 0.324), they contributed equally to the variation in mean arterial pressure (*p* value for difference = 0.5). Stroke volume (*p* value ≤ 0.002) and inv TAC (*p* value ≤ 0.001) contributed equally to the variation in systolic pressures (*p* value for difference ≥ 0.9). When stroke volume and inv TAC were entered in the same regression model (model *R*^2^^ ^= 0.752 to 0.765), they contributed equally to the variation in systolic blood pressures (*p* value for difference = 0.7). Stroke volume, TAC and SVR were similar (*p* value ≥ 0.5) and associated to the same extent with blood pressure measures in non-dialysis and dialysis CKD patients (*p* value for difference ≥ 0.1). In receiver operator characteristic curve analysis, elevated systolic blood pressure was determined by stroke volume (*p* value = 0.005) and inv TAC (*p* value = 0.03) but not SVR (*p* value = 0.8). The calculated power of the study was 0.999 based on *α* = 0.05.

**Conclusions:**

The present investigation suggests that both volume load and vascular mechanisms should be considered in the management of hypertension among patients with CKD. The extent and relative potential impact of volume load and vascular mechanisms on blood pressure measures are as large in non-dialysis compared to dialysis CKD patients.

## Introduction

Hypertension is both a major cause and consequence of chronic kidney disease (1–4). Accordingly, hypertension is known to occur in 70% of non-dialysis (5) and 60%–90% of dialysis CKD patients (6). Up to 87% of patients with CKD and hypertension experience inadequate blood pressure control (7). Hypertension in CKD patients is caused by structural changes and consequent increased sodium retention, activation of the renin-angiotensin-aldosterone and sympathetic nervous systems, endothelial dysfunction and the use of erythropoietin stimulating agents (8, 9). The contemporary view is that each of these causes engender increased blood pressure through vascular mechanisms including reduced aortic compliance and increased peripheral or systemic vascular resistance or/and extracellular fluid expansion (8–10). However, the pathophysiology of hypertension in chronic kidney disease remains markedly under investigated (11).

In population studies that were performed in high income countries, hypertension was found to be mediated by impaired vascular mechanisms rather than volume overload (12, 13). In fact, volume load is reportedly either unaltered or reduced in hypertension (10, 14). In CKD patients, marked arteriosclerosis that is particularly due to the replacement of elastin by collagen and aberrant bone mineral metabolism induced vascular calcification is highly prevalent (13). This results in substantially increased aortic stiffness (15). However, in addition to impaired vascular mechanisms, volume overload associates with blood pressure in both non-dialysis (16) and dialysis CKD patients (17). The prevalence of volume overload is ∼45% in non-dialysis (16) and 50% in dialysis CKD patients (17). Improved volume control through frequent or/and long dialysis sessions has a profound beneficial impact on hypertension in patients with end-stage renal disease or kidney failure (6, 17, 18).

Both steady state (mean arterial or distending pressure) and pulsatile (systolic, diastolic and pulse pressure) components of blood pressure impact cardiovascular risk in hypertension (10, 19–23). In patients with CKD, the most documented contributing blood pressure component to cardiovascular risk is high systolic blood pressure (24). The 2021 Kidney Disease: Improving Global Outcomes (KDIGO) guideline now recommend to target systolic blood pressure control in patients with CKD (24). Also, compared to brachial or peripheral blood pressures, aortic or central blood pressures are more strongly associated with cardiovascular events (25). Increased steady state or mean arterial pressure is particularly associated with chronic kidney disease progression (26, 27).

Improvement in our understanding of the pathophysiology of hypertension in CKD patients is needed to achieve more optimal and appropriate blood pressure control (11). In this regard, the relative contribution of volume overload and vascular mechanisms as well as their interactions to increases in blood pressure in patients with CKD is currently unknown. Also, to what extent the pathophysiology of hypertension differs in non-dialysis compared to dialysis CKD patients awaits elucidation. In the present study, we first assessed the confounder adjusted and mutually independent potential impact of volume load (stroke volume) and vascular mechanisms (total arterial compliance and systemic vascular resistance) on peripheral and central systolic blood pressure as well as mean arterial pressure in CKD patients. We subsequently determined whether the extent and potential effects of volume load and vascular mechanisms on blood pressure measures differ in non-dialysis compared to dialysis patients.

## Patients and methods

### Patients

The current study was performed in accordance with the Helsinki declaration as revised in 2013. The University of Witwatersrand Human (Medical) Research Committee approved the protocol (protocol number: M15-08-43). Each patient provided informed written consent prior to participation. The study design was reported previously (28–30). One hundred and fifteen patients participated. These comprised 67 non-dialysis and 48 dialysis patients. Patients with active infection or/and cancer, previously diagnosed heart failure and a Chronic Kidney Disease Epidemiology Collaboration estimated glomerular filtration rate (eGFR) of ≥60 ml/min/1.73 m^2^ were excluded.

### Baseline recorded characteristics

Methods that were employed in this study have been reported previously (28–30). Recorded baseline characteristics comprised demographic features, anthropometric measures, major traditional cardiovascular risk factors, non-traditional or renal cardiovascular risk factors and the use of cardiovascular drugs and erythropoietin stimulating agents. Established cardiovascular disease comprised ischemic heart disease (acute myocardial infarction, percutaneous transluminal coronary angioplasty and coronary artery bypass graft), cerebrovascular disease (stroke and transient ischemic attack) and peripheral vascular disease, the presence of which was confirmed by a cardiologist, neurologist and vascular surgeon, respectively. All investigations were performed on a single day. In dialysis patients, this was done on a day prior to a haemodialysis session. Dialysis was performed thrice weekly using 4-hour sessions. Most dialysis patients (72.9%) had an arteriovenous fistula (68.7%) or arteriovenous graft (4.2%). All patients were in sinus rhythm at the time of investigation.

### Hemodynamic characteristics

Mean arterial pressure for the peripheral waveform was determined electronically by the SphygmoCor device (see below) and using the formula

$$\mathrm{MP}=\frac{\sum_{i=T_{0}}^{T_{F}}P_{i}}{n}$$

where *T*_0 _= start of the waveform; *T_F_*_ _= end of the waveform; *P_i_*_ _= pressure points and *n* = number of pressure points.

Central systolic blood pressure and forward wave pressure were determined using a high-fidelity SPC-301 micromanometer (Miller instrument, Inc., Houston, Texas), interfaced with a computer using SpygmoCor software, version 9.0 (AtCor Medical Pty. Ltd., West Ryde, New South Wales, Australia), as previously reported (31, 32). After resting for 15 min in the supine position, arterial waveforms at the radial (dominant arm), carotid and femoral artery were recorded for a time period of ten consecutive waveforms (heart beats). Calibration of the pulse wave was done by manual measurement (auscultation) of the brachial blood pressure taken immediately prior to recordings. A validated generalized transfer function incorporated in the SphygmoCor software was used to convert the peripheral pressure waveform into a central aortic waveform. The results were discarded when systolic and diastolic variability of consecutive waveforms exceeded 5% or the amplitude of the pulse wave signal was less than 80 mV. All measurements were made by single experienced observer (CR) who was unaware of the cardiovascular risk factor profiles of the patients. Technically sound measurements of the central pressure wave were obtained in 109 patients. Brachial or peripheral blood pressure were recorded in all patients employing the oscillometric SunTech device (SunTech Medical, USA) (33). Recorded peripheral blood pressure in this study represents the average of ≥3 measurements taken at least 30 s apart after sitting quietly for at least five minutes. The optimal systolic blood pressure target in CKD is currently uncertain (34). In the present study, a systolic blood pressure of ≥130 mmHg was considered elevated.

Echocardiography was performed in accordance with the American Society of Echocardiography convention (35) and employing a Philips CX50 POC Compact CompactXtreme Ultrasound System [Philips Medical Systems (Pty) Ltd, USA] equipped with a 1.8–4.2 MHz probe that allowed for M-mode, 2-D and tissue Doppler measurements, as previously described (30). Patients were examined in the partial left decubitus position. We assessed left ventricular geometry and systolic (lateral s' and midwall fractional shortening as measures of longitudinal and circumferential myocardial contractility, respectively, and ejection fraction as an index of chamber or pump function) function.

Left ventricular dimensions were determined by measuring the left ventricular internal end diastolic and end systolic diameters and wall thickness (left ventricular septal and posterior wall thickness) in the parasternal long axis view by two-dimensional directed M-mode echocardiography. The Teichholz method was used to assess left ventricular end diastolic volume. Stroke volume was determined from the difference between left ventricular end diastolic and systolic volumes as evaluated upon employing the Z-derived method. Cardiac output was determined as stroke volume × heart rate. Left ventricular midwall fractional shortening was assessed using the previously reported formula as [(LVID_ed_ + 0.5 *H*_ed_) − LVID_es_ + 0.5 *H*_es_)]/LVID_ed_ + 0.5 *H*_ed_) where LVID is left ventricular internal diameter, *H* is wall thickness (mean of septal + posterior wall thickness), ed is end diastole and es is end systole (36, 37). Left ventricular ejection fraction was calculated as [(left ventricular end diastolic volume − left ventricular end systolic volume)/left ventricular end diastolic volume] × 100. Systemic vascular resistance was calculated as (mean arterial pressure − right atrial pressure)/cardiac output, assuming the right arterial pressure is 0 mmHg. Heart rate was determined from the length of an averaged peripheral waveform captured over a 10 s period, using the formula: 1,000/the length of an averaged peripheral waveform captured over a 10 s period ×60. Total arterial compliance (TAC) was calculated as stroke volume/central or aortic pulse pressure.

Echocardiographic measurements were made by the same observer that performed the arterial function evaluation. Intra-observer echocardiographic measurement variability is low in our setting with Pearson's correlation coefficients and variances (mean % difference (SD)) for left ventricular end-diastolic diameter, septal wall thickness, posterior wall thickness, E and e' of 0.92, 0.72, 0.76, 0.88 and 0.93 (*p* < 0.0001 for all), and −0.41 (4.16), 0.45 (7.74), 1.74 (6.08), 0.16 (9.95) and −1.46 (8.58), respectively.

### Data analysis

Results are expressed as mean (SD), median (interquartile range) or proportions as appropriate. Non-normally distributed variables were logarithmically transformed prior to entering them in linear multivariable regression models.

Data among subgroups including non-dialysis vs. dialysis patients and those with controlled vs. uncontrolled systolic blood pressure were compared in age, sex and black population origin adjusted models. Potential confounders for subsequent multivariate analysis were also identified in age, sex and black population origin adjusted models. Black population origin was included in regression models because the mean (SD) mean and central as well as peripheral systolic blood pressure were or tended to be consistently larger in black patients compared to those from other population origins (105 (14) mmHg vs. 100 (11) mmHg (*p*-Value = 0.06), 136 (22) mmHg vs. 127 (17) mm Hg (*p*-Value = 0.006) and 148 (24) mmHg vs. 137 (18) mmHg (*p*-Value = 0.004)), respectively.

Associations of stroke volume and vascular mechanisms (systemic vascular resistance and total arterial compliance) with mean and central and peripheral systolic blood pressure were assessed in confounder adjusted models. These relationships were also analysed in stratified analysis by dialysis status. They were also re-evaluated in a sensitivity analysis among patients without established cardiovascular disease. The associations of stroke volume and vascular mechanisms with elevated systolic blood pressure were determined in receiver operator characteristic (ROC) curve analysis.

As only 27.1% (*n* = 13) of dialysis patients did not have an arteriovenous fistula or graft, we did not compare the associations of stroke volume and vascular mechanisms with blood pressure measures in appropriately adjusted multivariate analysis between patients with and without the respective vascular access mode.

The data were analysed on IBM SPSS statistical program (version 27.0, IBM, USA) and Statistica 8.0 application package (version 14.0, TIBCO, USA). The power of this study was calculated using STATA (version 18, Stata Corp, USA).

## Results

### Recorded baseline characteristics in non-dialysis and dialysis patients

The baseline characteristics in non-dialysis and dialysis patients are shown in Table 1. Black patients were more frequently on dialysis, whereas white patients did more often not require dialysis. Dialysis patients exercised more frequently than their non-dialysis counterparts. Phosphate and parathyroid hormone concentrations were larger, whereas haemoglobin levels were lower in dialysis compared to non-dialysis patients. Dialysis patients used calcium channel blockers, diuretics and erythrocyte stimulating agents more often than those that were not on dialysis. Sodium-glucose cotransporter-2 inhibitors were not available in South Africa at the time of the study (2016) and hence not used in the present cohort.

**Table 1:** Baseline recorded characteristics in non-dialysis and dialysis patients.

Characteristics	Non-dialysis (*n* = 67)	Dialysis (*n* = 48)	*p-*Value
Demographics			
Age (years)	59.0 (13.8)	55.8 (14.3)	0.7
Female sex (%)	31.3	45.8	0.1
Black (%)	**28**.**4**	**56**.**2**	**0**.**005**
Asian (%)	31.3	22.9	0.2
White (%)	**35**.**8**	**8**.**3**	**0**.**003**
Mixed (%)	4.5	12.5	0.1
CKD duration (years)	6.0 (5.2)	4.6 (3.3)	0.3
Life style factors			
Alcohol use	1.5	2.1	0.8
Exercise	**25**.**4**	**52**.**1**	**0**.**003**
Anthropometry			
BMI (kg/m^2^)	27.8 (5.4)	26.8 (5.6)	0.5
Waist-hip ratio	0.96 (0.11)	0.98 (0.09)	0.2
Major traditional CV risk factors			
Hypertension (%)	86.6	95.8	0.8
Uncontrolled systolic blood pressure	74.6	79.2	0.7
Smoking (%)	4.5	0.0	–
Dyslipidemia (%)	85.5	71.4	0.2
Diabetes (%)	31.3	39.6	0.6
Non-traditional CV risk factors			
Dialysis duration (months)		24 (12–36)	
Estimated GFR (ml/min/1.73 m^2^)	35 (21)		
Phosphate (mmol/L)	**1.2 (0.5)**	**1.4 (0.6)**	**0**.**02**
Parathyroid hormone (pg/ml)	**83.0 (56.0–159.5)**	**502.8 (182.0–785.7)**	**<0**.**001**
Haemoglobin (g/dl)	**12.8 (2.8)**	**10.8 (9.7–12.0)**	**0**.**001**
Treatment			
Antihypertensive agent use (%)	86.6	95.8	0.3
Antihypertensives (*n*)	2.1 (1.3)	2.4 (1.1)	0.3
ACEI/ARB use (%)	80.3	80.4	1.0
Calcium channel blocker use (%)	**31**.**3**	**61**.**0**	**0**.**01**
Diuretic use (%)	36.4	31.2	0.3
Beta blocker use (%)	**37**.**9**	**56**.**5**	**0**.**04**
Alpha blocker use (%)	22.7	20.0	0.6
Statin use (%)	69.7	56.5	0.9
ESA use (%)	**16**.**4**	**89**.**6**	**<0**.**001**
**Cardiovascular disease (%)**	26.9	29.2	0.2

Data are expressed as mean (SD), median (interquartile range) or proportions and analysed in age, sex and black population origin adjusted regression models. Significant differences are shown in bold. CKD, chronic kidney disease; BMI, body mass index; GFR, glomerular filtration rate; ACEI, angiotensin converting enzyme inhibitors; ARB, angiotensin receptor blockers; ESA, erythropoietin-stimulating agents.

### Hemodynamic characteristics in non-dialysis and dialysis patients

The hemodynamic characteristics in non-dialysis and dialysis patients are given in Table 2. Central systolic blood pressure was larger in dialysis compared to non-dialysis patients. The other recorded hemodynamic characteristics did not differ among the two groups.

**Table 2:** Hemodynamic characteristics in non-dialysis and dialysis patients.

Characteristics	Non-dialysis (*n* = 67)	Dialysis (*n* = 48)	*p* value
Mean arterial pressure (mmHg)	100 (10)	105 (14.2)	0.07
Central systolic blood pressure (mmHg)	**127** (**17)**	**137** (**19)**	**0**.**007**
Peripheral systolic blood pressure (mmHg)	137 (19)	146 (23)	0.1
Central pulse pressure (mmHg)	44 (15)	49 (17)	0.2
Stroke volume (ml/beat)	69 (24)	71 (26)	0.6
Heart rate (beats/min)	73 (15)	77 (12.9)	0.6
Cardiac output (L/min)	4.9 (1.8)	5.5 (2.2)	0.3
SVR (mmHg/L per min)	21.1 (15.4–27.1)	20.4 (15.2–24.3)	0.5
TAC (ml/mmHg)	1.63 (1.33–2.11)	1.62 (1.14–1.96)	0.8
Pf (mmHg)	31.0 (10.2)	35.0 (11.0)	0.2
LV end diastolic volume (ml)	130 (61)	140 (68)	0.2
LV ejection fraction (%)	64.0 (14.3)	61.9 (14.6)	0.08
LV lateral wall s’ (cm/s)	8.8 (2.3)	8.0 (2.1)	0.2
LV midwall fractional shortening (%)	19.6 (4.8)	18.4 (5.6)	0.1

Data are expressed as mean (SD), median (interquartile range) or proportions and analysed in age, sex and black population origin adjusted regression models. Significant differences are shown in bold. SVR, systemic vascular resistance; Pf, forward wave pressure; TAC, total arterial compliance; LV, left ventricular.

### Associations of baseline characteristics with blood pressure measures at *p* value ≤ 0.1 in all, non-dialysis or/and dialysis patients

Data analysis aimed at identifying potential confounders for subsequent multivariate analysis was performed in all and non-dialysis as well as dialysis patients. Table 3 shows that age, sex, black population origin, exercising status, diabetes, haemoglobin levels and erythrocyte stimulating agents were each associated at *p* value ≤ 0.1 with one or more blood pressure measures in all, non-dialysis or/and non-dialysis patients.

**Table 3:** Associations of baseline characteristics with blood pressure measures at *p* value ≤ 0.1.

All CKD patients				Non-Dialysis CKD patients (*n* = 67)			Dialysis CKD patient (*n* = 48)		
Characteristics	MAP	CSBP	PSBP	MAP	CSBP	PSBP	MAP	CSBP	PSBP
Age	−0.026; 0.8	**0.189; 0.05**	0.106; 0.3	0.164; 0.2	**0.325; 0.009**	**0.202; 0.1**	−**0.218; 0.1**	0.043; 0.8	0.185; 0.2
Female	−0.123; 0.2	0.028; 0.8	−0.052; 0.6	−**0.339; 0.006**	−0.169; 0.2	−0.173; 0.2	0.058; 0.7	0.200: 0.2	0.185; 0.2
Black PO	**0.175; 0.06**	**0.265; 0.006**	**0.271; 0.004**	0.093; 0.5	**0.251; 0.04**	**0.265; 0.006**	0.155; 0.3	0.208; 0.2	**0.265; 0.006**
Exercise	**0.198; 0.04**	**0.169; 0.08**	**0.144; 0.1**	0.169; 0.2	0.107; 0.4	0.007; 1.0	0.166; 0.4	0.070; 0.7	0.166; 0.3
Diabetes	−0.008; 0.9	0.112; 0.2	**0.155; 0.1**	0.077; 0.5	0.122; 0.3	0.234; 0.06	−0.003; 1.0	0.165; 0.3	0.105; 0.5
Haemoglobin	0.001; 1.0	−0.074; 0.4	−0.118; 0.2	−0.129; 0.4	0.014; 0.9	0.037; 0.8	−0.129; 0.4	−0.167; 0.3	−**0.376; 0.01**
ESA use	**0.173; 0.07**	**0.224; 0.02**	**0.219; 0.02**	−**0.016; 0.9**	**0.146; 0.3**	0.056; 0.7	**0.323; 0.03**	0.168; 0.3	**0.356; 0.02**

Results are expressed as partial R; (*p*-Value). Data were analysed in age, sex and black population origin adjusted regression models. Significant relationships are shown in bold. CKD, chronic kidney disease; MAP, mean arterial pressure; CSBP, Central systolic blood pressure; PSBP, peripheral systolic blood pressure; PO, population origin; ESA, erythrocyte stimulating agents.

### Potential determinants of mean arterial pressure in CKD patients

**Figure 1:**
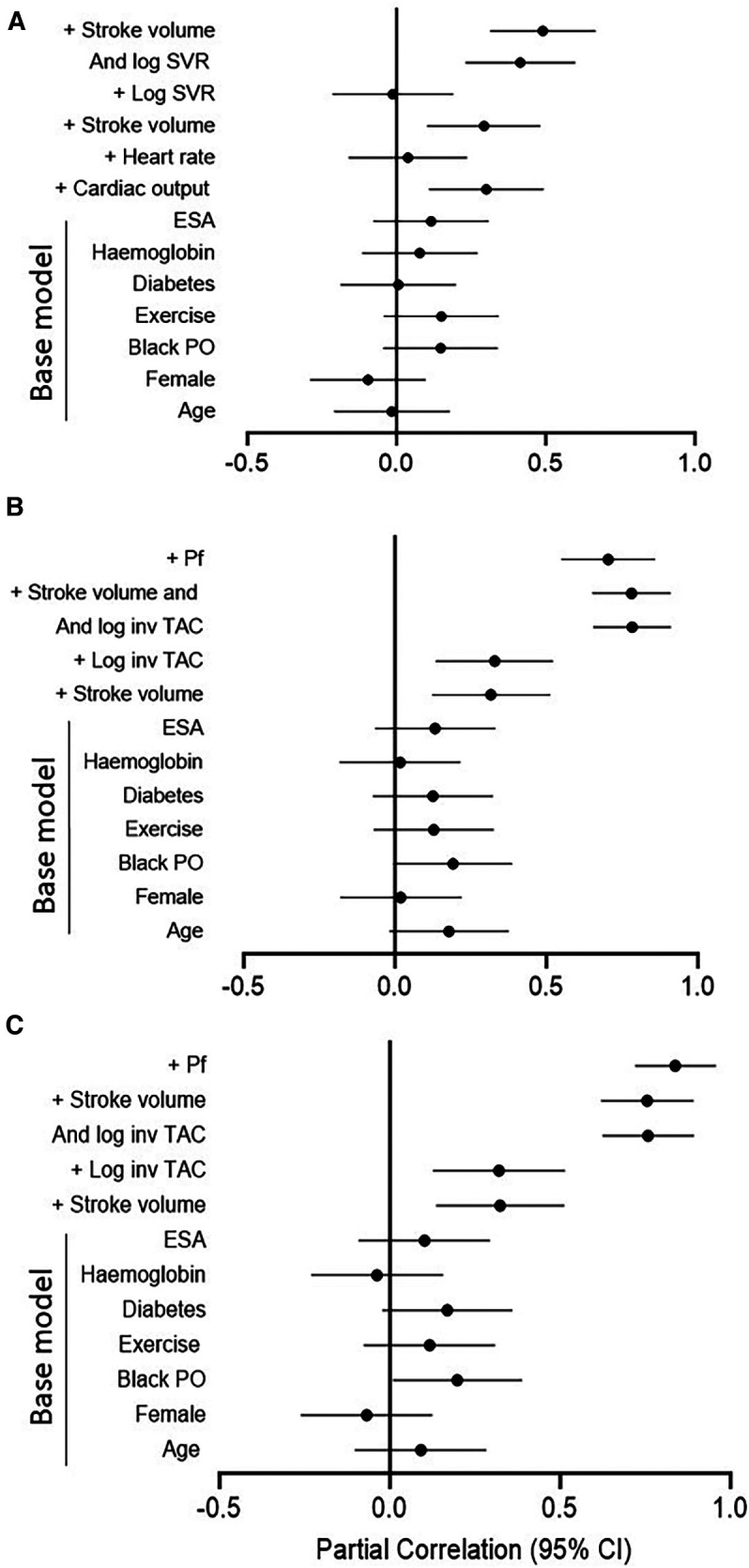
Partial correlations for the models in Table 4 (**A**) and Table 5 (**B** for central systolic blood pressure and **C** for peripheral systolic blood pressure). Log, logarithmically transformed; SVR, systemic vascular resistance; ESA, erythrocyte stimulating agents; PO, population origin; Pf, forward wave pressure; TAC, total arterial compliance.

Table 4 gives the relative contributions of cardiac output, stroke volume and systemic vascular resistance to the variation in mean arterial blood pressure in all included patients. None of the baseline characteristics were independently associated with mean arterial pressure (first model). Baseline characteristics explained only 10.5% (model *R*^2 ^= 0.105) of the variation in mean arterial pressure. When cardiac output (second model) or stroke volume (fourth model) were added to the model, both these characteristics were independently associated with mean arterial pressure and the model *R*^2^ increased to 0.190 and 0.179, respectively. When stroke volume was replaced by stroke volume indexed to body surface area in the fourth model, the results were unaltered (model *R*^2 ^= 0.190, standardised *β* = 0.280, *p* value = 0.004). By contrast, neither heart rate (third model) nor systemic vascular resistance (fifth model) was related to mean arterial pressure and, accordingly, the model *R*^2^ remained unchanged upon entering these characteristics. When systemic vascular resistance was re-calculated on the assumption that right atrial pressure was 10 mmHg (30) in the fifth model, the results were unaltered (model *R*^2 ^= 110, standardised *β* = 0.020, *p* value = 0.8). When stroke volume and systemic vascular resistance were entered into the same model (sixth model), both characteristics were strongly and to a similar extent associated with mean arterial pressure. The respective model *R*^2^ increased to 0.324. In this regard, stroke volume and systemic vascular resistance were strongly interrelated (Spearman's rho = −0.797, *p* value < 0.001). Upon entering stroke volume x systemic vascular resistance without (seventh model) and with (eighth model) the individual interaction terms, the model *R*^2^ remained unaltered. In the latter model, only the interaction between stroke volume and systemic vascular resistance tended to be associated (*p* value = 0.05) with mean arterial pressure. The partial correlations (95% confidence intervals) for the relationships in Table 4 are shown in Figure 1A.

**Table 4:** Potential determinants of mean arterial pressure in CKD patients.

Characteristics	Cumulative *R*^2^	*β* (SE)	*p* value	Std. β
	0.105			
Baseline = Age and		−0.014 (0.088)	0.9	−0.016
Female and		−2.429 (2.446)	0.3	−0.096
Black PO and		3.865 (2.517)	0.1	0.154
Exercise and		4.110 (2.639)	0.1	0.161
Diabetes and		0.149 (2.546)	0.9	0.006
Haemoglobin and		0.432 (0.536)	0.4	0.091
ESA		3.571 (2.980)	0.2	0.145
+Cardiac output	0.190	**0.002** (**0.001)**	**0**.**002**	**0**.**313**
+Heart rate	0.106	0.033 (0.088)	0.7	0.039
+Stroke volume	0.179	**0.145** (**0.047)**	**0**.**003**	**0**.**286**
+Log SVR	0.110	−0.946 (7.388)	0.9	−0.013^a^
+Stroke volume and	0.324	**0.450** (**0.081)**	**<0**.**001**	**0**.**870**
Log SVR		**51.498** (**11.465)**	**<0**.**001**	**0**.**716**^b^
+Stroke volume x SVR	0.326	**19.931** (**3.551)**	**<0**.**001**	**0**.**504**
+Stroke volume and	0.334	0.146 (0.178)	0.4	0.282
log SVR and		8.989 (24.925)	0.7	0.125
Stroke volume x SVR		15.496 (8.097)	0.05	0.392

Data were analysed in multivariate regression models. Significant associations are shown in bold. CKD, chronic kidney disease; β, regression coefficient; SE, standard error; std., standardised; PO, population origin; ESA, erythrocyte stimulating agent; log, logarithmically transformed; SVR, systemic vascular resistance.

^a^ *p* value = 0.03 versus relation with stroke volume in the preceding model.

^b^ *p* value = 0.5 versus relation with stroke volume in the same model.

Based on the sample size of 115 patients, *α* = 0.05 and 10 covariates in multiple regression models, the calculated power of the study was 0.999. This was established using the associations with mean arterial pressure.

### Potential determinants of central and peripheral systolic blood pressure in CKD patients

Table 5 gives the relative contributions of stroke volume and the inverse of TAC (inv TAC) to the variation in central and peripheral systolic blood pressure in all included patients. Among the baseline characteristics, only black population origin tended to be (*p* value = 0.05) associated and was related to (*p* value = 0.04) central and systolic blood pressure, respectively. In the respective models, baseline characteristics explained ∼16% of the variation in systolic blood pressures. When stroke volume or inv TAC were added to the model, both these characteristics were independently associated with systolic blood pressures and the model *R*^2^ increased to 0.246 to 0.266. When stroke volume and inv TAC were entered into the same model, both characteristics were strongly and to a similar extent associated with systolic blood pressures. The respective model *R*^2^ increased to 0.708 for central systolic blood pressure and 0.685 for peripheral systolic blood pressure. In this regard, stroke volume and inv TAC were strongly interrelated (Spearman correlation coefficient = −0.581, *p* value < 0.001). Upon entering stroke volume x inv TAC without and with the individual interaction terms, the model *R*^2^ increased further from 0.752 to 0.771. In the latter models, only the interaction between stroke volume and inv TAC were associated with systolic blood pressures (*p* value < 0.001 for both). In parallel to these findings, Pf was strongly associated with systolic blood pressures with a model *R*^2^ of 0.910 and 0.929 for central and peripheral systolic blood pressure, respectively. The partial correlations (95% confidence intervals) for the relationships in Table 5 are shown in Figures 1B,C.

**Table 5:** Potential determinants of central and peripheral systolic blood pressure in CKD patients.

Central systolic blood pressure					Peripheral systolic blood pressure			
Characteristics	Cumulative *R*^2^	β (SE)	*p v* alue	Std. β	Cumulative *R*^2^	β (SE)	*P* value	Std. β
Baseline=	0.161				0.159			
Age and		0.249 (0.137)	0.07	0.179		0.138 (0.147)	0.3	0.091
Female and		0.763 (3.915)	0.8	0.019		−2.867 (4.068)	0.5	−0.066
Black PO and		7.865 (4.045)	0.05	0.195		**8.721** (**4.185)**	**0**.**04**	**0**.**202**
Exercise and		5.381 (4.163)	0.2	0.132		5.304 (4.389)	0.2	0.121
Diabetes and		5.106 (4.053)	0.2	0.123		7.467 (4.233)	0.08	0.168
Haemoglobin and		0.141 (0.839)	0.9	0.019		−0.349 (0.892)	0.7	−0.043
ESA		6.340 (4.756)	0.2	0.162		5.217 (4.955)	0.3	0.124
+Stroke volume	0.246	**0.254** (**0.078)**	**0**.**002**	**0**.**308**	0.254	**0.276** (**0.080)**	**<0**.**001**	**0**.**314**
+Log inv TAC	0.252	**34.475** (**10.165)**	**<0**.**001**	**0**.**332**^a^	0.266	**35.881** (**10.879)**	**0**.**001**	**0**.**320**^b^
+Stroke volume and	0.708	**0.806** (**0.066)**	**<0001**	**0**.**978**	0.685	**0.835** (**0.075)**	**<0**.**001**	**0**.**937**
Log inv TAC		**106.225** (**8.703)**	**<0**.**001**	**1**.**023**^c^		**110.152** (**9.774)**	**<0**.**001**	**0**.**982**^d^
+Stroke volume X Inv TAC	0.765	**1.121** (**0.072)**	**<0**.**001**	**0**.**911**	0.752	**1.177** (**0.080)**	**<0**.**001**	**0**.**886**
+Stroke volume and	0.771	0.123 (0.148)	0.4	0.149	0.756	0.046 (0.165)	0.7	0.051
log Inv TAC and		8.118 (20.937)	0.7	0.078		−3.104 (23.33**)**	0.9	−0.028
Stroke volume X Inv TAC		**1.017** (**0.202)**	**<0**.**001**	**0**.**827**		**1.174** (**0.225)**	**<0**.**001**	**0**.**886**
+Pf	0.910	**0.681** (**0.075)**	**<0**.**001**	**0**.**367**	0.929	**1.013** (**0.071)**	**<0**.**001**	**0**.**507**

Data were analysed in multivariate regression models. Significant associations are shown in bold. CKD, chronic kidney disease; β, regression coefficient; SE, standard error; std., standardised; PO, population origin; ESA, erythrocyte stimulating agent; log, logarithmically transformed; log, logarithmically transformed; inv, inverse of; TAC, total arterial compliance; Pf, forward wave pressure.

^a^ *p* value = 0.9 versus relation with stroke volume in the preceding model.

^b^ *p* value = 1.0 versus relation with stroke volume in the preceding model.

^c^ *p* value = 0.7 versus relation with stroke volume in the same model.

^d^ *p* value = 0.7 versus relation with stroke volume in the same model.

Based on the sample size of 115 patients, *α* = 0.05 and 10 covariates in multiple regression models, the calculated power was 0.999. This was established using the associations with both central and peripheral blood pressure.

### Potential impact of stroke volume and vascular mechanisms on blood pressure measures in non-dialysis and dialysis patients

**Figure 2:**
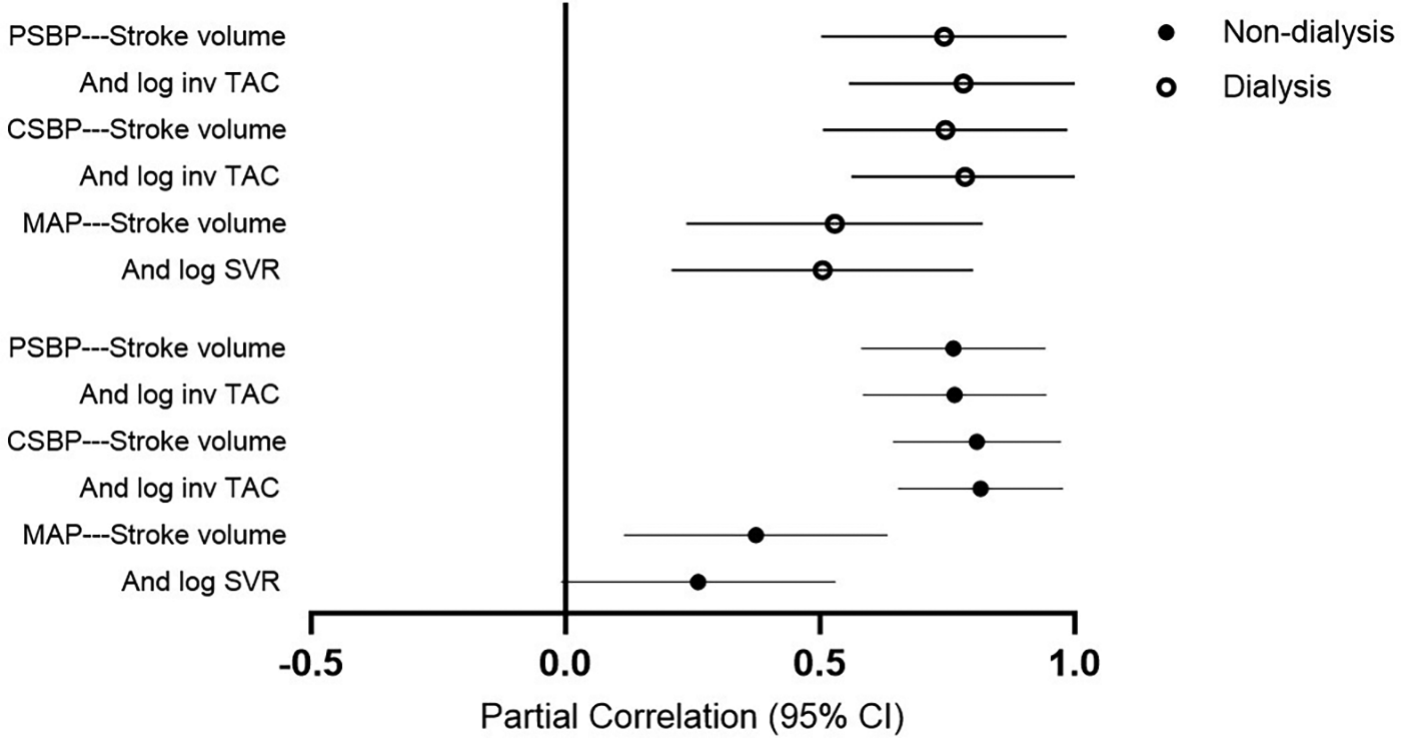
Partial correlations for the models in Table 6. PSBP, peripheral systolic blood pressure; log, logarithmically transformed; inv, inverse of; TAC, total arterial compliance; CSBP, central systolic blood pressure; MAP, mean arterial pressure.

Table 6 gives the mutually independent impact of stroke volume and vascular mechanisms on the variation in blood pressure measures in confounder adjusted stratified analysis by dialysis status. The relative contribution of stroke volume and vascular mechanisms (systemic vascular resistance and inv TAC) to the variation in mean arterial blood pressure and central as well as peripheral systolic blood pressure were assessed in models that included both characteristics. The relative potential impact of stroke volume and vascular mechanisms on blood pressure measures was similar in each of these models. Also, the potential impact of stroke volume and vascular mechanisms on blood pressure measures was consistently similar in non-dialysis and dialysis patients. The partial correlations (95% confidence intervals) for the relationships in Table 6 are shown in Figure 2.

**Table 6:** Potential impact of stroke volume and vascular mechanisms on blood pressure measures in non-dialysis compared to dialysis patients.

Non-dialysis patients (*n* = 67)					Dialysis patients (*n* = 48)			
Mean arterial pressure								
Characteristics	*R* ^2^	β (SE)	*p* value	Std. β	*R* ^2^	β (SE)	*p v* alue	Std. β
Stroke volume and	0.345	**0.294** (**0.101)**	**0**.**005**	**0**.**633**	0.417	**0.543** (**0.148)**	**<0**.**001**	**0**.**991**^c^
Log SVR		**25.842** (**13.283)**	**0**.**05**	**0**.**406**^a^		**75.433** (**21.824)**	**0**.**001**	**0**.**976**^b,d^
Central systolic blood pressure								
Stroke volume and	0.770	**0.797** (**0.081)**	**<0**.**001**	**1**.**053**	0.696	**0.776** (**0.123)**	**<0**.**001**	**0**.**903**^g^
Log inv TAC		**97.503** (**9.615)**	**<0**.**001**	**1**.**049**^e^		**122.813** (**17.146)**	**<0**.**001**	**1**.**094**^f,h^
Peripheral systolic blood pressure								
Stroke volume and	0.705	**0.874** (**0.103)**	**<0**.**001**	**1**.**021**	0.718	**0.760** (**0.121)**	**<0**.**001**	**0**.**863**^k^
Log inv TAC		**105.230** (**12.316)**	**<0**.**001**	**1**.**000**^i^		**119.911** (**16.921)**	**<0**.**001**	**1**.**043**^j,l^

Data were analysed in age, sex, black population origin, exercise status, diabetes, haemoglobin and erythropoietin stimulating agents adjusted regression models. Significant associations are shown in bold. β, regression coefficient; SE, standard error; SVR, systemic vascular resistance; inv, inverse of, TAC, total arterial compliance.

^a^ *p* value = 0.4 versus relation with stroke volume in the same model.

^b^ *p* value = 1.0 versus relation with stroke volume in the same model.

^c^ *p* value = 0.3 versus relation with stroke volume in non-dialysis CKD patients.

^d^ *p* value = 0.1 versus relation with log SVR in non-dialysis CKD patients.

^e^ *p* value = 1.0 versus relation with stroke volume in the same model.

^f^ *p* value = 0.4 versus relation with stroke volume in the same model.

^g^ *p* value = 0.4 versus relation with stroke volume in non-dialysis CKD patients.

^h^ *p* value = 0.8 versus relation with log inv TAC in non-dialysis CKD patients.

^i^ *p* value = 0.9 versus relation with stroke volume in the same model.

^j^ *p* value = 0.4 versus relation with stroke volume in the same model.

^k^ *p* value = 0.4 versus relation with stroke volume in non-dialysis CKD patients.

^l^ *p* value = 0.9 versus relation with log inv TAC in non-dialysis CKD patient.

### Impact of stroke volume and vascular mechanisms on blood pressure measures in patients without established cardiovascular disease

Table 7 gives the mutually independent potential impact stroke volume and vascular mechanisms on blood pressure measures in confounder adjusted models among patients without established cardiovascular disease [*n* = 83 (72.2%)]. The relative contribution of stroke volume and vascular mechanisms (systemic vascular resistance and inv TAC) to the variation in mean arterial blood pressure and central as well as peripheral systolic blood pressure were assessed in models that included both characteristics. The respective relationships were similar to those in all patients (Tables 4, 5).

**Table 7:** Potential impact of stroke volume and vascular mechanisms on blood pressure measures in CKD patients without established cardiovascular disease.

Mean arterial pressure				
Characteristics	Model *R*^2^	β (SE)	*P* value	Std. β
Stroke volume and	0.421	**0.439** (**0.087)**	**<0**.**001**	**0**.**734**
Log SVR		**57.194** (**13.265)**	**<0**.**001**	**0**.**622**
Central systolic blood pressure				
Stroke volume and	0.767	**0.801** (**0.076)**	**<0**.**001**	**0**.**863**
Log inv TAC		**111.822** (**9.523)**	**<0**.**001**	**0**.**968**
Peripheral systolic pressure				
Stroke volume and	0.729	**0.816** (**0.087)**	**<0**.**001**	**0**.**831**
Log inv TAC		**114.809** (**10.874)**	**<0**.**001**	**0**.**939**

Data were analysed in age, sex, black population origin, exercise status, diabetes, haemoglobin and erythropoietin stimulating agents adjusted regression models. Significant associations are shown in bold. CKD, chronic kidney disease; β, regression coefficient; SE, standard error; SVR, systemic vascular resistance; inv, inverse of, TAC, total arterial compliance.

### Potential determinants of stroke volume

Stroke volume is a valuable marker of volume effects on systemic flow and hence volume status (38, 39). However, stroke volume is reportedly determined by not only cardiac preload (left ventricular end diastolic volume), but also left ventricular contractility and cardiac afterload (38, 39). Cardiac afterload (systemic vascular resistance and the inverse of TAC) was included in our multivariate regression models in Tables 4–7. Table 8 shows the contribution of left ventricular end diastolic volume, contractility (left ventricular midwall fractional shortening and lateral s') and pump function (ejection fraction) to the variation in stroke volume in confounder adjusted regression models. Based on the variable *R*^2 ^s in the models, left ventricular end diastolic volume, midwall fractional shortening and ejection fraction explained 39.4%, 16.1% and 12.5% of the variation in stroke volume. Hence, left ventricular end diastolic volume contributed 2.4 fold more than left ventricular midwall fractional shortening and 3.1 fold more than ejection fraction to the variation in stroke volume. Left ventricular lateral s' was not associated with stroke volume.

**Table 8:** Potential determinants of stroke volume in CKD patients.

Characteristics	Model *R*^2^	β (SE)	Std. β	*p* value	Variable *R*^2^
LV end diastolic volume	0.461	**0.245** (**0.030)**	**0**.**649**	**<0**.**001**	**0**.**394**
LV midwall fractional shortening (%)	0.364	**1.664** (**0.447)**	**0**.**361**	**<0**.**001**	**0**.**161**
LV lateral s’	0.113	0.672 (1.045)	0.063	0.5	0.004
Ejection fraction	0.221	**0.614** (**0.161)**	**0**.**356**	**<0**.**001**	**0**.**125**

Data were analysed in age, sex, black population origin, exercise status, diabetes, haemoglobin and erythropoietin stimulating agents adjusted regression models. Significant associations are shown in bold. CKD, chronic kidney disease; β, regression coefficient; SE, standard error; LV, left ventricular.

In view of these findings and in order to confirm that the potential impact of stroke volume on blood pressure measures is not attributable to left ventricular contractility and pump function, the confounder adjusted and mutually independent relationships of stroke volume and vascular mechanisms with blood pressure parameters as given in Tables 4, 5, were re-evaluated in regression models that included left ventricular midwall fractional shortening or ejection fraction as additional potential confounders. Table 9 shows that the associations of stroke volume (as well as systemic vascular resistance and the inverse of TAC) with blood pressure parameters were unaltered and independent of left ventricular function measures.

**Table 9:** Associations of stroke volume and vascular mechanisms with blood pressure parameters after additional adjustment for left ventricular contractility (midwall fractional shortening) and performance (ejection fraction) in CKD patients.

Mean arterial pressure				
Characteristics	Model *R*^2^	β (SE)	*p* value	Std. β
Stroke volume and	0.472	**0.496** (**0.88)**	**<0**.**001**	**0**.**956**
Log SVR and		**51.389** (**12.346)**	**<0**.**001**	**0**.**669**
Midwall fractional shortening		−0.472 (0.243)	0.06	−0.199
Stroke volume and	0.382	**0.485** (**0.076)**	**<0**.**001**	**0**.**937**
Log SVR and		**51.962** (**10.924)**	**<0**.**001**	**0**.**722**
Ejection fraction		−**0164** (**0.082)**	**0**.**04**	−**0**.**186**
Central systolic blood pressure				
Stroke volume and	0.756	**0.779** (**0.075)**	**<0**.**001**	**0.944**
Log inv TAC and		**101.575** (**9.624)**	**<0**.**001**	**0**.**900**
Midwall fractional shortening		−0.201 (0.260)	0.4	−0.053
Stroke volume and	0.714	**0.818** (**0.067)**	**<0**.**001**	**0**.**992**
Log inv TAC and		**104.311** (**8.774)**	**<0**.**001**	**1**.**005**
Ejection fraction		−0.124 (0.091)	0.2	−0.089
Peripheral systolic pressure				
Stroke volume and	0.729	**0.829** (**0.084)**	**<0**.**001**	**0**.**938**
Log inv TAC and		**101.061** (**10.849)**	**<0**.**001**	**0**.**836**
Midwall fractional shortening		−0.440 (0.294)	0.1	−0.108
Stroke volume and	0.692	**0.849** (**0.075)**	**<0**.**001**	**0**.**953**
Log inv TAC and		**107.768** (**9.831)**	**<0**.**001**	**0**.**961**
Ejection fraction		−0.155 (0.101)	0.1	−0.101

Data were analysed in age, sex, black population origin, exercise status, diabetes, haemoglobin and erythropoietin stimulating agents adjusted regression models. Significant associations are shown in bold. CKD, chronic kidney disease; β, regression coefficient; SE, standard error; log, logarithmically transformed; SVR, systemic vascular resistance; inv, inverse of, TAC, total arterial compliance.

### Baseline characteristics in patients with normal and elevated systolic blood pressure

Table 10 gives the baseline characteristics in patients with normal and elevated systolic blood pressure. Eighty-eight (76.4%) of the patients had elevated systolic blood pressure. Systolic blood pressure was below 120 mmHg in only 13 (11.3%) of study participants. Age and CKD duration were associated with elevated systolic blood pressure. Black patients experienced elevated systolic blood pressure more often, whereas the reverse applied to Asian patients. Patients who exercised had more frequently elevated systolic blood pressure. Patients with elevated systolic blood pressure used calcium channel blockers and statins more frequently and less often had established cardiovascular disease.

**Table 10:** Baseline recorded characteristics in CKD patients without and with elevated systolic blood pressure.

Characteristics	Normal SBP (*n* = 27)	Elevated SBP (*n* = 88)	*p* value
Demographics			
Age (years)	**52.3 (16.4)**	**59.3 (12.2)**	**0**.**02**
Female sex (%)	51.9	32.9	0.2
Black (%)	**25**.**9**	**44**.**3**	**0**.**03**
Asian (%)	**51**.**8**	**20**.**5**	**0**.**004**
White (%)	18.5	26.1	0.9
Mixed (%)	3.7	9.1	0.4
CKD duration (years)	**4.5 (4.1)**	**5.7 (4.6)**	**0**.**04**
Life style factors			
Alcohol use	0.0	2.3	1.0
Exercise	**11**.**1**	**44**.**3**	**0**.**004**
Anthropometry			
BMI (kg/m^2^)	27.7 (6.7)	27.3 (5.1)	1.0
Waist-hip ratio	0.95 (0.07)	0.97 (0.11)	0.9
Major traditional CV risk factors			
Hypertension (%)	85.2	92.1	0.8
Smoking (%)	0.0	3.4	–
Dyslipidemia (%)	73.9	81.5	0.5
Diabetes (%)	29.6	36.4	0.8
Non-traditional CV risk factors			
Dialysis	37.0	43.2	0.4
Dialysis duration (months)	25.4 (19.7)	31.1 (23.8)	0.7
Estimated GFR (ml/min/1.73 m^2^)	37 (19)	34 (21)	0.7
Phosphate (mmol/L)	1.4 (0.4)	1.3 (0.6)	0.7
Parathyroid hormone (pg/ml)	104.0 (56.0–534.0)	186.0 (71.4–520.7)	0.8
Haemoglobin (g/dl)	11.5 (1.9)	12.2 (2.8)	0.4
Treatment			
Antihypertensive agent use (%)	85.2	92.0	0.8
Antihypertensives (*n*)	1.7	2.3	0.1
ACE/ARB use (%)	77.8	81.2	0.8
Calcium channel blocker use (%)	**22**.**2**	**50**.**0**	**0**.**01**
Diuretic use (%)	29.6	35.6	0.5
Beta blocker use (%)	37.0	48.2	0.3
Alpha blocker use (%)	11.1	25.0	0.4
Statin use (%)	**44**.**4**	**70**.**6**	**0**.**009**
ESA use (%)	29.6	52.3	0.07
Cardiovascular disease (%)	**37**.**0**	**25**.**0**	**0**.**03**

Data are expressed as mean (SD), median (interquartile range) or proportions and analyzed in age, sex and black population origin adjusted regression models. Significant differences are shown in bold. SVR, systemic vascular resistance; TAC, total arterial compliance; LV, left ventricular.

### Hemodynamic characteristics in normal and elevated systolic blood pressure

Table 11 shows the hemodynamic characteristics in normal and elevated systolic blood pressure. As expected, all blood pressure measures were larger in patients with elevated systolic blood pressure. Stroke volume, cardiac output and left ventricular end diastolic volume were larger, whereas TAC was smaller in patients with uncontrolled elevated systolic blood pressure. By contrast, systemic vascular resistance was similar in both groups. Pf was larger in patients with uncontrolled elevated systolic blood pressure.

**Table 11:** Hemodynamic characteristics in CKD patients with and without elevated systolic blood pressure.

Characteristics	Normal SBP (*n* = 27)	Elevated SBP (*n* = 88)	*p v*alue
Mean arterial pressure (mmHg)	**89** (**6)**	**106** (**11)**	**<0**.**001**
Central systolic blood pressure (mmHg)	**115** (**19)**	**135** (**17)**	**<0**.**001**
Peripheral systolic blood pressure (mmHg)	**116** (**7)**	**149** (**18)**	**<0**.**001**
Central pulse pressure (mmHg)	**35** (**9)**	**54** (**15)**	**<0**.**001**
Stroke volume (ml/beat)	**57** (**23)**	**74** (**23)**	**0**.**01**
Heart rate (beats/min)	75 (18)	75 (13)	0.8
Cardiac output (L/min)	**4.2** (**1.7)**	**5.5** (**2.0)**	**0**.**01**
SVR (mmHg/L per min)	22.8 (15.9–30.1)	19.9 (15.4–26.2)	0.2
TAC (ml/mmHg)	**1.81** (**1.36–2.64)**	**1.52** (**1.15–1.92)**	**0**.**008**
Pf (mmHg)	**23.9** (**6.7)**	**35.6** (**10.2)**	**<0**.**001**
LV end diastolic volume (ml)	**116** (**58)**	**152** (**65)**	**0**.**003**
LV ejection fraction (%)	62.9 (17.0)	63.2 (13.65)	0.6
LV lateral wall s’ (cm/s)	8.8 (2.7)	8.4 (2.1)	0.6
LV midwall fractional shortening (%)	18.7 (4.8)	19.3 (5.2)	0.7

Data are expressed as mean (SD), median (interquartile range) or proportions and analysed in age, sex and black population origin adjusted regression models. Significant differences are shown in bold. SVR, systemic vascular resistance; TAC, total arterial compliance; LV, left ventricular.

### Hemodynamic correlates of elevated systolic blood pressure

**Figure 3:**
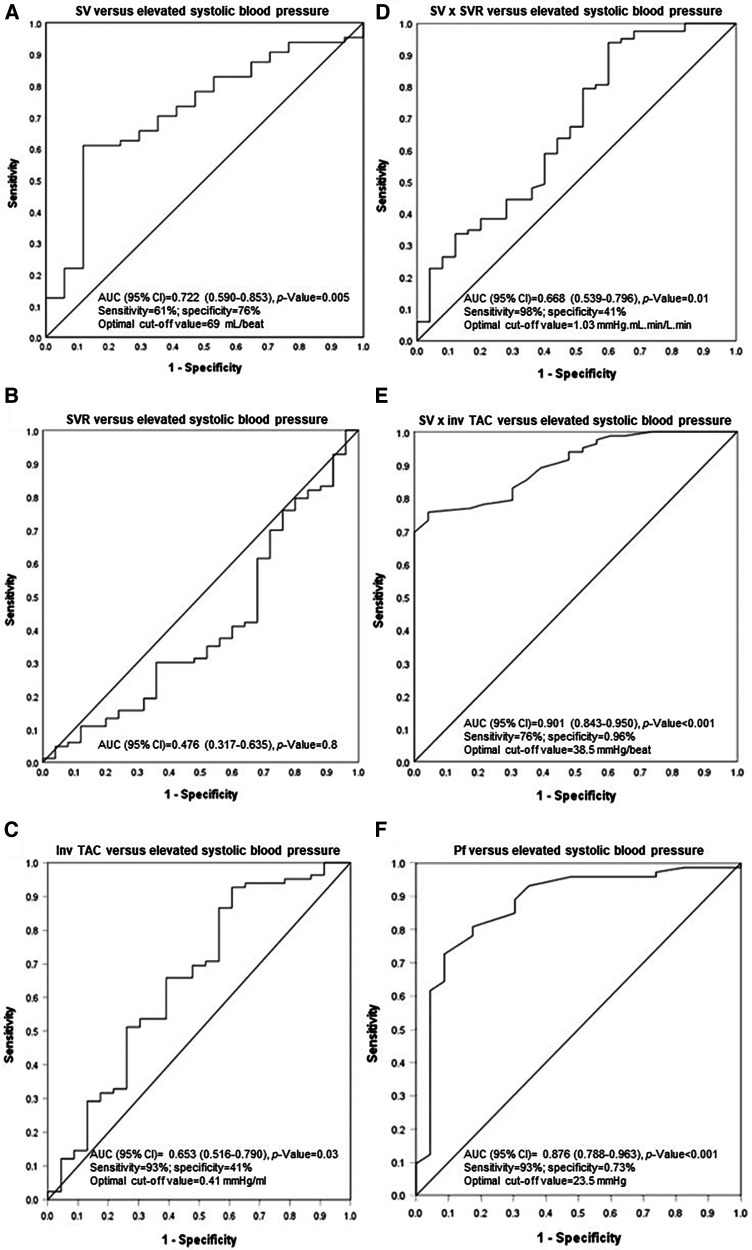
Associations of stroke volume and vascular mechanisms with uncontrolled systolic blood pressure. SV, stroke volume; SVR, systemic vascular resistance; inv, inverse of; TAC, total arterial compliance; Pf, forward wave pressure.

The hemodynamic correlates of elevated systolic blood pressure were assessed in ROC curve analysis. As given in Figure 3, stroke volume (AUC (95% CI) = 0.722 (0.590–0.853)) and inv TAC (AUC (95% CI) = 0.653 (0.590–0.853)) were associated with uncontrolled elevated systolic blood pressure whereas systemic vascular resistance was not (AUC (95% CI) = 0.476 (0.317–0.635)). Compared to stroke volume x inv TAC (AUC (95% CI) = 0.901 (0.843–0.950)), stroke volume x systemic vascular resistance was more weakly related to elevated systolic blood pressure (AUC (95% CI) = 0.668 (0.539–0.796)). The stroke volume x inv TAC association with elevated systolic blood pressure was paralleled by an equally strong relationship between Pf and elevated systolic blood pressure. Table 12 shows the associations of hemodynamic characteristics with elevated systolic blood pressure in stratified analysis by dialysis status. The respective relationships were consistently similar in non-dialysis and dialysis patients.

**Table 12:** Comparison of the associations of stroke volume and vascular mechanisms with elevated systolic blood pressure between non-dialysis and dialysis patients.

Non-dialysis CKD patients (*n* = 67)			Dialysis CKD patients (*n* = 48)	
Characteristics	AUC (95% CI)	*p*-Value	AUC (95% CI)	*p* value
Stroke volume	**0.732** (**0.713–0.982)**	**0**.**005**	0.636 (0.863–1.000)^a^	0.07
Log SVR	0.457 (0.291–0.624)	0.6	0.315 (0.084–0.545)^b^	0.09
Log inv TAC	0.626 (0.462–0.791)	0.1	0.653 (0.377–0.929)^c^	0.2
Stroke volume × Log SVR	**0.699** (**0.523–0.867)**	**0**.**02**	0.616 (0.451–0.817)^d^	0.3
Stroke volume × Log inv TAC	**0.906** (**0.833–0.978)**	**<0**.**001**	0.882 (0.774–0.981)^e^	0.3
Pf	**0.848** (**0.713–0.982)**	**<0**.**001**	**0.936** (**0.863–1.000)**^f^	**0**.**002**

Data were analysed in receiver operator characteristic curve analysis. Significant associations are shown in bold. CKD, chronic kidney disease; AUC, area under the curve; SVR, systemic vascular resistance; inv, inverse of; TAC, total arterial compliance; Pf, forward wave pressure.

^a^ *p* value = 0.8 versus relations with stroke volume in non-dialysis CKD patients.

^b^ *p* value = 0.3 versus relations with log SVR in non-dialysis CKD patients.

^c^ *p* value = 0.9 versus relations with log inv TAC in non-dialysis CKD patients.

^d^ *p* value = 0.9 versus relations with stroke volume x log SVR in non-dialysis CKD patients.

^e^ *p* value = 0.7 versus relations with stroke volume x log inv TAC in non-dialysis CKD patients.

^f^ *p* value = 0.2 versus relations with stroke volume in non-dialysis CKD patients.

## Discussion

The present multiethnic study examined for the first time the relative potential contribution of stroke volume and vascular mechanisms to mean and central as well as peripheral systolic blood pressures in non-dialysis and dialysis CKD patients. Vascular mechanisms comprised systemic vascular resistance and TAC. The hypertension burden was large in the present CKD cohort with 90.4% of study participants being affected. The main novel findings in this study are sixfold. Firstly, in fully adjusted regression models, stroke volume contributed at least as much as vascular mechanisms to the variation in mean and central and peripheral systolic blood pressures. Secondly, stroke volume, systemic vascular resistance and TAC did not differ between non-dialysis and dialysis CKD patients. Thirdly, the relative contribution of stroke volume and vascular mechanisms to variation in mean and central and peripheral systolic blood pressures were as large in non-dialysis compared to dialysis patients. Fourthly, elevated systolic blood pressure was as prevalent in non-dialysis as in dialysis CKD patients. Fifthly, in ROC curve analysis, stroke volume and the inverse of TAC were directly associated with uncontrolled systolic blood pressure; by contrast, systemic vascular resistance was not related to uncontrolled systolic blood pressure. Sixthly, the relationships of stroke volume and the inverse of TAC with uncontrolled systolic blood pressure was similar in non-dialysis and dialysis patients. The calculated power of the study was 0.999. The present findings have implications in the management of hypertension among CKD patients.

In the population at large, increased mean arterial pressure is mostly determined by elevated systemic vascular resistance whereas enhanced pulsatile pressures are generally attributable to reduced aortic elasticity (8). Stroke volume and cardiac output are typically unaltered or even reduced in primary hypertension (10, 14). However, recent studies revealed an association of volume overload with increases in blood pressure among dialysis as well as non-dialysis CKD patients (16, 17). Compared to conventional haemodialysis, frequent and prolonged haemodialysis is more effective at controlling hypertension (6, 17, 18). An interaction between volume load and vascular mechanisms and hence forward wave pressure ultimately determines pulsatile pressures (10, 12, 40). In this regard, the most striking findings in the present study were identified in multivariate regression models. In contrast to findings in the general population where vascular mechanisms mediate hypertension (12, 13), in confounder adjusted models, stroke volume contributed to the same extent as inv TAC to the variation in central and peripheral systolic blood pressures in the present CKD cohort. Stroke volume and inv TAC were inversely related. Upon entering both characteristics in the same model to determine their mutually independent potential impact, stroke volume and inv TAC contributed similarly to the variation in systolic blood pressures and the model *R*^2^ increased markedly. The latter finding suggested that stroke volume and inv TAC can interact in the mediation of systolic blood pressures. Indeed, when the interaction term stroke volume x inv TAC was entered in a separate model, the model *R*^2^ increased further and, when the individual interaction terms (stroke volume and TAC) were added as independent variables to the model, they were no longer related to systolic blood pressures. Taken together, volume load contributes as much as vascular mechanisms to systolic blood pressure in CKD patients.

Volume load and systemic vascular resistance reportedly interact upon mediating steady state or mean arterial pressure (10). In this regard, in confounder adjusted models, cardiac output and stroke volume were strongly associated with mean arterial blood pressure. By contrast, systemic vascular resistance was unrelated to mean arterial blood pressure. Stroke volume and systemic vascular resistance were inversely related. When both characteristics were entered in the same model, their contributions to the variation in mean arterial pressure were similar. This was accompanied by an increase in the model R^2^, which suggested that there was an interaction between both characteristics. Accordingly, the interaction term stroke volume x systemic vascular resistance explained as much as both mutually independent characteristics of the variation in mean arterial pressure and, when the individual interaction terms were entered in the same model, they were unrelated to mean arterial pressure. All in all, our results indicate that volume load contributes more or at least as much as systemic vascular resistance to the variation in mean or arterial blood pressure. In sensitivity analyses among patients without cardiovascular disease, the potential impact of stroke volume and vascular mechanisms on blood pressure measures remained consistent.

Current guidelines on the management of hypertension in CKD patients emphasize the need for adequate volume control particularly in patients on dialysis (41). Importantly in the present context, the present investigation revealed that stroke volume as a marker of volume load as well as vascular mechanisms were impaired to the same extent in non-dialysis as in dialysis patients. Equally and likely more pertinent, the potential impact of volume load and vascular mechanisms on blood pressure measures was as strong in non-dialysis as in dialysis patients. Our findings suggest that adequate volume load control through reduced salt and fluid intake is as if not more important in non-dialysis compared to dialysis patients with CKD, this particularly given that, per definition, dialysis as a measure of volume control is not used in the former group.

In this study, more than three quarters of participants had a brachial systolic blood pressure of ≥130 mmHg despite the overall use of an average of 2.2 antihypertensives. The 2021 KDIGO guideline recommends a systolic blood pressure target of below 120 mmHg (24). In the present investigation, systolic blood pressure was below 120 mmHg in only 11% of study participants. Patients with elevated systolic blood pressure had a larger volume load as represented by increased left ventricular end diastolic volume, stroke volume and cardiac output as well as more impaired TAC compared to those with normal systolic blood pressure. By contrast, systemic vascular resistance was similar in both groups. Stroke volume (AUC (95% CI) for ROC curve = 0.722 (0.590–0.853)) and inv TAC (AUC (95% CI) for ROC curve = 0.653 (0.516–0.790)) and particularly the interaction between these characteristics (AUC (95% CI) for ROC curve = 0.901 (0.843–0.950)) were strongly associated with elevated systolic blood pressure. By contrast, systemic vascular resistance was not related to elevated systolic blood pressure. These findings were similar in non-dialysis and dialysis CKD patients. The current results reiterate the critical importance of volume control in the management of hypertension in not only dialysis but also non-dialysis patients with CKD.

Intriguingly and seemingly paradoxically, in this study, patients with uncontrolled systolic blood pressure exercised more frequently and had less prevalent established cardiovascular disease compared to their controlled systolic blood pressure counterparts. However, we believe that this is due to a survival bias as patients with uncontrolled blood pressure together with comorbid cardiovascular disease or/and concurrent adverse lifestyle factors would be expected to more likely have died prior to enrolment in the study.

Overall, the prevalence of hypertension in the present study may be larger than that reported in non-dialysis (86.6% vs. 70%) (5) and dialysis CKD patients (95.8% vs. 60% to 90%) (6) that live in high income countries. This is likely due to the large proportion (40%) of CKD patients from black population origin in the current cohort (29). The present study has several limitations. Firstly, our cross-sectional study design precludes drawing inferences on the direction of causality. Secondly, we used office blood pressure measurements. Ambulatory blood pressures may be more strongly associated with incident cardiovascular disease in CKD patients (24, 41). Thirdly, peak aortic flow and aortic characteristic impedance may be more strongly implicated than stroke volume and TAC, respectively, in mediating pulsatile pressures (12, 13). However, peak aortic flow reportedly approximates stroke volume and the inverse of aortic characteristic impedance and TAC are strongly correlated (*R* = 0.83) (41). In line with the latter, proximal aortic stiffness that is due to the replacement of elastin by collagen and vascular calcification accounts for half of inv TAC (42, 43). Also, in a recent population study, we found that TAC was as strongly associated with pulsatile pressures as was characteristic impedance (44). Additionally, the relationships of stroke volume and proximal aortic flow with pulsatile pressures were similar (44). In the present study, together with potential confounders, stroke volume and inv TAC as well as their interaction explained between 68% and 77% of the variation in systolic blood pressures. In parallel to this, forward wave pressure together with potential confounders explained 91% to 93% of systolic blood pressures. Except for an inconsistent relationship with black population origin, the potential confounders that were entered in the respective models were not independently associated with mean arterial and systolic blood pressure. Moreover, the included potential confounders explained only 10.5% of the variation in mean arterial pressure and 15.9% to 16.1% of systolic blood pressures. Whether longitudinal investigations with the inclusion of proximal aortic flow and aortic characteristic impedance can further our understanding of the pathophysiology that underlies CKD induced hypertension merits further study. Fourthly, potentially important contributors to blood pressure measures in CKD that were not recorded in the present study also encompass sodium intake as estimated by natriuresis and, among dialysis patients, preserved urinary output and dry weight. In this investigation, weight was recorded only at the time of the study. The respective characteristics should be included in future investigations that are aimed at determining the pathophysiology of hypertension in patients with CKD. Lastly, although none of the patients had symptoms or physical signs that were attributed to heart failure by the involved nephrologist (H-C H) at the time of the study, a recent investigation revealed that heart failure with preserved ejection fraction is not only the most common (35%) but also the most frequently overlooked (69%) heart failure phenotype in patients on haemodialysis (45). Our patients all underwent a previous evaluation by cardiologists but this was not systematically done at the time of the study. Hence, it is possible if not likely that a proportion of patients with established cardiovascular disease as represented by previous myocardial infarction and coronary angioplasty or bypass grafting had heart failure with preserved ejection fraction in the present cohort. Nevertheless, in a sensitivity analysis in which patients with established cardiovascular disease were excluded, the relationships of stroke volume and vascular mechanisms with blood pressure measures were unaltered.

In conclusion, independent of one another, stroke volume contributes as much as systemic vascular resistance to the variation in mean arterial or distending pressure and as much as inv TAC to the variation in systolic blood pressures in patients with CKD. Stroke volume and vascular mechanisms may interact upon mediating blood pressure measures in CKD patients. The potential impact of stroke volume and vascular mechanisms on blood pressures is as strong in non-dialysis patients as in dialysis patients. Stroke volume and inv TAC but not systemic vascular resistance is strongly associated with elevated systolic blood pressure and this applies as much in non-dialysis as in dialysis patients. Our results suggest that both volume load and vascular mechanisms should be considered in the management of hypertension among patients with CKD. The extent and relative potential impact of volume load and vascular mechanisms on blood pressure measures are as large in non-dialysis compared to dialysis CKD patients.

## Data Availability

The original contributions presented in the study are included in the article/Supplementary Materials, further inquiries can be directed to the corresponding author.
